# Cell Death Triggers Induce MLKL Cleavage in Multiple Myeloma Cells, Which may Promote Cell Death

**DOI:** 10.3389/fonc.2022.907036

**Published:** 2022-07-28

**Authors:** Jing Chen, Shiyu Wang, Bart Blokhuis, Rob Ruijtenbeek, Johan Garssen, Frank Redegeld

**Affiliations:** ^1^ Division of Pharmacology, Utrecht Institute for Pharmaceutical Sciences, Faculty of Science, Utrecht University, Utrecht, Netherlands; ^2^ Genmab B.V., Utrecht, Netherlands; ^3^ Nutricia Research, Utrecht, Netherlands

**Keywords:** MLKL, caspases, necroptosis, proteolytic cleavage, multiple myeloma, bortezomib, EPA, DHA

## Abstract

Necroptosis is a type of caspase-independent programmed cell death that has been implicated in cancer development. Activation of the canonical necroptotic pathway is often characterized with successive signaling events as the phosphorylation of mixed lineage kinase domain-like (MLKL) by receptor-interacting protein kinase-3 (RIPK3), followed by MLKL oligomerization and plasma membrane rupture. Here, we demonstrate that omega-3 polyunsaturated fatty acids DHA/EPA and the proteasome inhibitor bortezomib induce necroptosis in human multiple myeloma (MM) cells in a RIPK3 independent manner. In addition, it seemed to be that phosphorylation of MLKL was not essential for necroptosis induction in MM cells. We show that treatment of MM cells with these cytotoxic compounds induced cleavage of MLKL into a 35 kDa protein. Furthermore, proteolytic cleavage of MLKL was triggered by activated caspase-3/8/10, and mutation of Asp140Ala in MLKL blocked this cleavage. The pan-caspase inhibitor ZVAD-FMK efficiently prevented DHA/EPA and bortezomib induced cell death. In addition, nuclear translocation of total MLKL and the C-terminus were detected in treated MM cells. Collectively, this present study suggests that caspase-mediated necroptosis may occur under (patho)physiological conditions, delineating a novel regulatory mechanism of necroptosis in RIPK3-deficient cancer cells.

## Introduction

Multiple myeloma (MM), the second most frequent hematological malignancy, is still considered as an incurable disease due to its highly heterogenous genomic and phenotypic nature, high-risk, therapeutic refractoriness, disease relapse and acquisition of drug resistance (1). The outcomes of MM have improved markedly over the past decades with the introduction of several novel therapeutic agents including proteasome inhibitors such as bortezomib and carfilzomib; monoclonal antibodies such as elotuzumab and daratumumab; and immunomodulatory drugs such as thalidomide, lenalidomide, and pomalidomide. Since the proteasome inhibitor bortezomib was approved for treatment of refractory/relapsed MM patients, prominent clinical outcome has been seen, although moderate side-effects were reported occasionally (2). Combinational use of bortezomib and other anti-cancer reagents has been suggested as well as useful strategy to improve the therapy outcome. Omega-3 polyunsaturated fatty acids (PUFAs) docosahexaenoic acid (DHA) and eicosapentaenoic acid (EPA) showed direct cytotoxic effects in various cancer types including multiple human solid tumors, leukemia, lymphomas and MM (3, 4). Previously, it was demonstrated that administration of DHA or EPA prior to bortezomib can enhance the chemosensitivity in human MM cell lines (5). Although the synergistic effect was significant, the underlying mechanisms of action is still not clear. Based on the critical role of necroptosis in tumorigenesis and tumor development, the role of necroptosis in the anti-cancer effects of bortezomib and EPA/DHA in MM was further investigated.

Necroptosis is another type of programmed cell death in addition to apoptosis, morphologically characterized by cell swelling, plasma membrane rupture and release of intracellular contents. It has been implicated in numerous human pathological conditions, including viral and bacterial infections, neurodegenerative disorders, inflammatory disease, kidney injury and especially in multiple cancers (6). Necroptosis can be initiated by multiple cytokines, pathogen- and damage-associated molecular patterns, of which TNFα-induced necroptosis was mostly studied. The binding of TNF-α to TNFR1 triggers the formation of a membrane-associated complex consisting of TRADD, TRAF2/5, cIAP1/2, LUBAC and RIPK1. Within this complex, cIAP1/2 and LUBAC induce polyubiquitination of RIPK1, eventually leading to the activation of pro-survival signaling pathways such as nuclear transcription factor-kappa B (NF-κB) and mitogen-activated protein kinase (MAPK). Under specific conditions, such as in the presence of IAP antagonists or deubiquitinases, TRADD and RIPK1 are dissociated from TNFR1, which results in activation of caspase-8-dependent apoptosis (7). When caspase-8 is absent or inactivated and RIPK3 and MLKL are present in adequately high levels, RIPK1 together with RIPK3 and MLKL form a necroptosis-inducing complex, termed as necrosome. Within necrosome, RIPK1 and RIPK3 bind to each other through their RIP homotypic interaction motif (RHIM) and recruit MLKL to the complex, subsequently leading to MLKL activation by RIPK3-mediated phosphorylation at Thr357 and Ser358. Then, phosphorylated-MLKL undergoes conformational changes that triggers its subsequent unfolding and oligomerization. MLKL oligomer is able to translocate to the plasma membrane where it exerts pore-forming function to induce necroptosis (8). As such, MLKL is the executioner of necroptosis while RIPK3 is the key upstream kinase of MLKL that provides the necroptotic signal. Noteworthily, the necroptotic pathway is almost fully eliminated in many human malignancies, including colon cancer, melanoma and myeloid leukemia, due to the down-regulated expression level or promoter methylation-mediated inactivation of RIPK3 (9–11).

The human MLKL consists of an N-terminal four-helical bundle domain (NTD) (1-125 aa), a brace region (BR) with two α-helices (126-167 aa and 168-180 aa) and a C-terminal kinase like domain (KLD) (181-471 aa) (12). Recent studies demonstrated that the N-terminal domain NTD was required and sufficient to induce the formation of MLKL oligomers and further necroptosis in human and murine cells (13, 14). Interestingly, NTD with one α-helix of BR (1-167 aa) was found to be incapable of activating necroptosis, but NTD with complete BR (1-180 aa) was able to induce necroptosis (13). This insight suggested the critical role of the BR in triggering necroptosis. However, until now, there is no supportive evidence whether the NTD of MLKL is endogenously present under (patho)physiological conditions. Furthermore, the activated-MLKL was suggested to translocate to nucleus due to exposure of the C-terminal nuclear localization sequence (NLS, 224−256 aa) after phosphorylation, which was found to be not required for necroptosis but can facilitate cell death (15, 16).

In this study, it was demonstrated that MLKL, the effector protein of necroptosis, can be cleaved at Asp140 by active caspase-3/8/10 in MM cells. After cleavage, the N-terminal fragment (1-140 aa) might oligomerize itself and execute necroptosis while the C-terminal fragment (141-471 aa) undergoes degradation by proteasome. Additionally, the phosphorylation sites (Thr357 and Ser358) were not required for the cleavage. Moreover, DHA/EPA and bortezomib induce nuclear translocation of total MLKL and the C-terminus in MM cells, further suggesting their capacity to induce necroptosis. From these findings, an alternative necroptotic pathway may be inferred in RIPK3-deficient cancer cells, for example in MM cells, where necroptosis may be executed by N-terminal cleavage of MLKL when caspases are activated.

## Materials and Methods

### Reagents and Antibodies

DHA (D2534) and EPA (E2011) were purchased from Sigma. DHA and EPA were dissolved in ethanol to produce a 100 mM stock solution. Annexin V apoptosis detection kit (88-8005-74) was obtained from Thermo Fisher. Bortezomib was obtained from LC laboratories and dissolved in DMSO. Pan-caspase inhibitor Z-VAD-FMK (tlrl-vad) was purchased from *In vivo*Gen. Caspase-3 inhibitor Z-DEVD-FMK (FMK004), caspase-8 inhibitor Z-IETD-FMK (FMK007), caspase-10 inhibitor Z-AEVD-FMK (FMK009) and MG132 (1748) were obtained from R&D systems. Bis(sulfosuccinimidyl) suberate (BS^3^) (21586) were obtained from Thermo Scientific Pierce Biotechnology. Anti-Flag M2 Magnetic Beads was obtained from Sigma-Aldrich. Antibodies for MLKL (#14993), RIPK1 (#3493), RIPK3 (#13526), phospho-Ser358-MLKL (#91689), phospho-Ser166-RIPK1 (#65746), phospho-Ser227-RIPK3 (#93654), cleaved-caspase3 (#9661) and Flag M2 antibody (#2368) were purchased from Cell Signaling Technology; anti-GAPDH (sc-47724) was obtained from Santa Cruz Biotechnology. Anti-MLKL antibody (GTX107538) for immunoprecipitation was obtained from GeneTex (Hsinchu, Taiwan). Horse radish peroxidase-conjugated secondary antibodies were purchased from Dako Agilent.

### Cell Culture

All human multiple myeloma cell lines were maintained in RPMI-1640 medium, which was supplemented with 10% fetal calf serum (FCS), 2 mM L-glutamine, 100 IU penicillin, and 100 mg/ml streptomycin. HT-29 cell line was cultured in McCoy’s 5A modified medium supplemented with 10% FCS, 100 IU penicillin, and 100 mg/ml streptomycin. A549 cell line was maintained in F-12K Medium with 10% FCS. Human mast cells were obtained from surplus autologous stem cell concentrates (17) as previously described.

### MLKL Oligomerization

The formation of MLKL oligomers was studied by chemical crosslinking using a membrane-impermeable crosslinker bissulfosuccinimidyl suberate (BS^3^) which is commonly used for crosslinking of membrane proteins. MLKL oligomers was analyzed as described previously with minor modifications (18). Briefly, cells were washed twice with ice-cold PBS and cross-linked in PBS containing 2.5 mM BS^3^ for 1 h at 4°C. The crosslinking reaction was quenched by incubation with 40 mM Tris (pH 7.5) for 15 minutes at room temperature. Then, cells were washed twice with ice-cold PBS and then lysed with a lysis buffer [20 mM HEPES (pH 7.5), 150 mM NaCl, 1 mM Na_2_EDTA, 1 mM EGTA, 1% TritonX-100, 2.5 mM sodium pyrophosphate, 1 mM β-glycerophosphate, 1 mM Na_3_VO_4_ supplemented with protease inhibitor] for 30 min at 4°C. Cell lysates were collected by centrifugation at 4°C for 10 minutes at 12 000 rpm. MLKL oligomers were analyzed by non-reducing SDS–PAGE followed by western blotting with anti-MLKL antibody.

### Plasmids and Transfection

Constructs encoding C-terminally Flag tagged-MLK^WT^ (PPL01031-2a) and C-terminally Flag tagged-MLKL^T357A/S358A^ (PPL01031-2b) were purchased from Bioworld Technology. Point mutations were generated by the GeneArt™ Site-Directed Mutagenesis System (A13282, Thermo Fisher). All plasmids were verified by DNA sequencing. For transient expression, MM cells were transfected with 5 µg of indicated plasmids using Amaxa Nucleofector Device with cell line specific Nucleofector Kit C according to manufacturer’s instructions. After drug treatment, transfected cells were harvested and subjected to immunoprecipitation for cleavage study.

### Cell Death Measurement

FITC-conjugated Annexin V and Propidium iodide (PI) staining was performed to detect dead cells according to manufacturer’s protocol. Briefly, cells were seeded in triplicate into a 12-well plate at density of 200 × 10^3^ cells/well and exposed with indicated concentrations of drugs. After treatment, cells were collected by centrifugation at 1500 rpm for 5 min, washed once with PBS and once with binding buffer. Cells were resuspended in 100 µl binding buffer and stained with 5 µl Annexin V-FITC antibody for 15 minutes at room temperature in dark. Then, cells were washed once with binding buffer and resuspended in 100 µl binding buffer, then 2.5 µl of PI was then added and incubated in dark at 4°C for 5 minutes. The percent of dead cells (Annexin V^+^ PI^-/+^) were determined by a BD FACS Canto II flow cytometer.

### Immunoprecipitation

Immunoprecipitation of endogenous MLKL using anti-C-terminal MLKL antibody (GTX107538, GeneTex): approximately 20 ×10^6^ MM cells were treated with 500 nM bortezomib for 24 h and then lysed in 500 µl lysis buffer [0.5% TritonX-100 in PBS supplemented with protease inhibitor cocktail (Sigma-Aldrich)]. The cell lysates were centrifuged at 12 000 rpm for 15 min, and the supernatants were subjected to immunoprecipitation. Anti-MLKL antibody was crosslinked to protein G Dynabeads (10003D, Thermo Fisher) using the crosslinking reagent BS^3^. Then, 1.5 mg protein was incubated with the antibody crosslinked-beads overnight at 4°C, and then beads were washed 3 times with lysis buffer. The immunoprecipitates were eluted off the beads with a low pH elution buffer (0.1 M Glycine-HCl, pH 2.3). Acid elution was immediately neutralized using 1/10 volume of 1 M Tris-HCl (pH 7.5). The immunoprecipitated proteins were subsequently analyzed by western blotting.

Immunoprecipitation of exogenous Flag-tagged MLKL using anti-Flag M2 magnetic beads (M8823, Merck): 5 × 10^6^ MM cells were transfected with 5 µg of pcDNA3.1(+)-MLKL (WT)-C-term Flag vector for 18 h. Whole cell lysates were prepared using lysis buffer [50 mM Tris-HCl, pH 7.4, 150 mM NaCl, 1 mM EDTA, 1% TritonX-100, including protease inhibitors (Sigma-Aldrich)]. The cell lysates were incubated with anti-Flag M2 magnetic beads overnight at 4°C. The following day, beads were washed 3 times with lysis buffer, and the immunoprecipitants were eluted off the beads as described above. The immunoprecipitated proteins were subsequently analyzed by western blotting using anti-Flag antibody and anti-MLKL antibody.

### Western Blotting

Whole cell lysates were prepared with RIPA buffer [#89900 Thermo Fisher, 1× protease inhibitor cocktail (Roche), 1 × Phosphatase Inhibitor Cocktail (Roche)] on ice for 30 minutes. Then, cell lysates were collected after centrifugation at 4°C for 10 minutes at 12 000 rpm. Protein concentration was determined by BCA assay (Pierce BCA Protein Assay Kit; Thermo Fisher). Equal amounts of protein were subjected to reducing SDS-PAGE and then electrotransferred onto a polyvinylidene difluoride (PVDF) membrane. Membranes were blocked for 1 h in blocking buffer [TBST buffer containing 5% (w/v) skimmed milk] and probed with the specific primary antibodies in blocking buffer overnight at 4°C. Membranes were then washed three times with TBST (10 minutes each time) followed by incubation with relevant HRP-conjugated secondary antibody for 1 hour at room temperature. After washing with TBST (10 minutes, 3 times), membranes were developed using ECL (#1705061; Biorad) and detected by the ChemiDoc System (Bio-Rad).

### Nuclear Translocation Analysis

Nuclear translocation analysis by subcellular fractionation: nuclear fraction was collected as described previously (19). Briefly, cells were lysed on ice for 30 min using a lysis buffer containing 10 mM HEPES pH7.5, 1.5 mM MgCl2, 10 mM KCl, 0.5% NP40 and protease inhibitor. The supernatant was the cytoplasmic fraction. After three times washing with cold PBS, the pellet was further lysed with RIPA buffer for 30 min on ice and then centrifuged at 12 000 rpm for 10 min at 4°C. The supernatant was the nuclear fraction. Western blotting was performed to determine MLKL nuclear translocation.

Nuclear translocation analysis by immunocytochemical analysis: cells were cytocentrifuged on slides, fixed with 4% paraformaldehyde in PBS, permeabilized with 0.1% Triton X-100 for 10 min at room temperature and blocked with 5% goat serum in 1% BSA in PBS for 30 min. Then, cells were incubated with primary MLKL antibody in a humidified chamber overnight at 4°C and stained with AlexaFluor 594-conjugated secondary antibody at room temperature for 1 h in dark. Cells were mounted with ProLong Gold Antifade Mountant with DAPI for nuclear staining. The MLKL appeared red under fluorescence microscopy and the nuclei appeared blue. The red MLKL and blue nucleus were merged to purple fluorescence in the colocalization areas using Image J software.

### Mass Spectrometric Analysis

IP-Flag immunoprecipitants were resolved by SDS-PAGE and proteins were stained with Coomassie stain (LC6060, Thermo Fisher). Then, total MLKL and cleaved-MLKL bands were excised and de-stained followed by in-gel trypsin digestion. The peptides were extracted and analyzed on an UHPLC 1290 system (Agilent) coupled to an Orbitrap Q Exactive HF mass spectrometer (Thermo Fisher Scientific) as previously described (20).

### RNA Sequencing

Total RNA was extracted from OPM2 cells using RNeasy Mini Kit (74104, Qiagen) and RNA integrity was determined by Agilent 2100 analysis. RNA integrity numbers (RIN) of all samples were greater than 9.0. RNA-seq libraries were prepared and sequenced at Novogene (Novogene (UK) Company Limited) (21). Data were analyzed using Novosmart software.

### Statistics

Western blot image signal intensity was quantified using Image Lab 5.2.1. Statistical analysis was carried out using GraphPad Prism. Statistical significance was analyzed using one-way ANOVA. *P <* 0.05 was considered significant. Sequence alignment of BR of MLKL was performed using Jalview software (version 2.11.1.0).

## Results

### Necroptosis Is Involved in DHA/EPA and Bortezomib-Induced Cell Death in MM Cells

**Figure 1:**
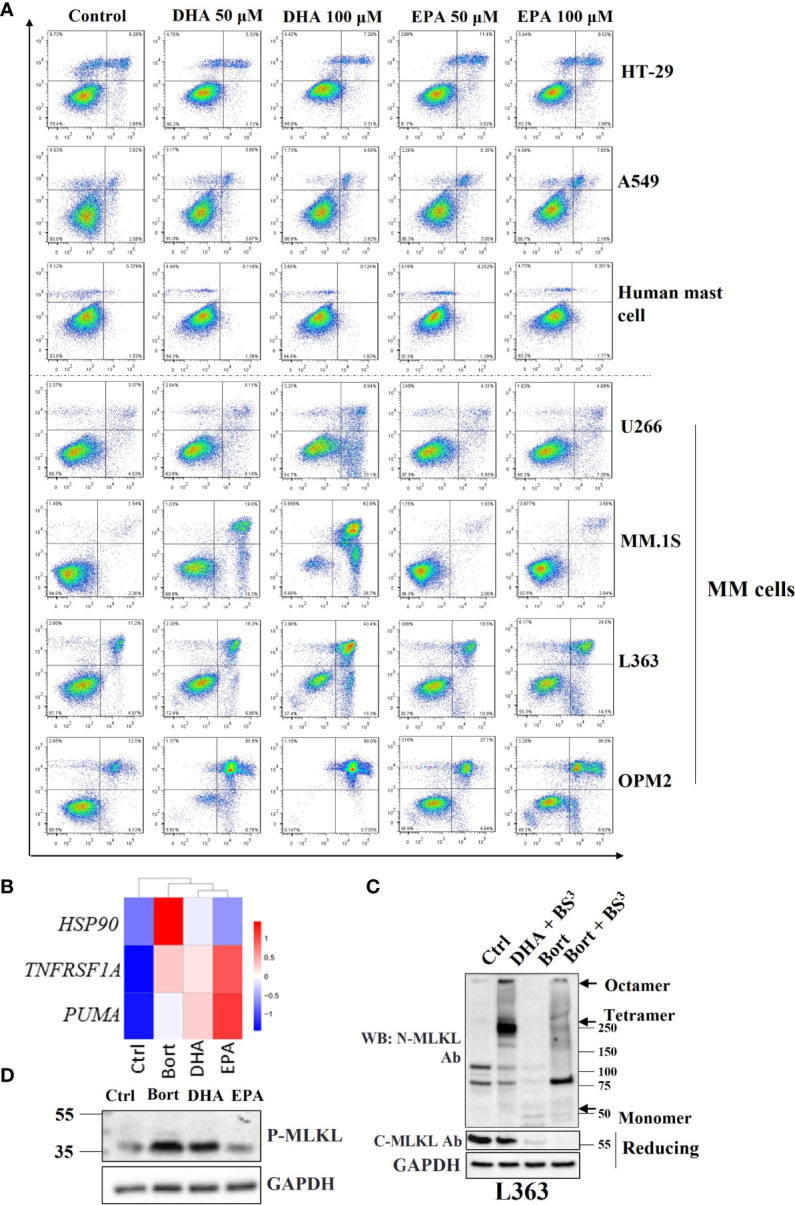
Necroptosis is involved in DHA/EPA and bortezomib-induced cell death in MM cells. **(A)** Cells were treated with 50 µM and 100 µM of DHA or EPA for 24 h. Cell death was determined by Annexin V and PI staining. **(B)** OPM2 cells were treated with 50 µM of DHA/EPA or 10 nM of bortezomib. RNA sequencing was performed. The expression levels of *HSP90*, *TNFRSF1A* and *PUMA* were shown in heatmap. **(C)** L363 cells were treated with DHA (50 µM) or bortezomib (500 nM) for 24 h and lysed after 1 h incubation with crosslinker BS^3^. Cell lysates were subjected to SDS-PAGE under non-reducing and reducing conditions and immunoblotted with indicated antibodies. Arrows indicate the oligomers of MLKL. **(D)** Cells were treated with 100 µM of DHA or EPA and 500 nM of bortezomib for 24 h and lysed with RIPA buffer. Whole cell lysates were subjected to western blotting with antibodies against p-MLKL and GAPDH.

It was demonstrated recently that omega-3 fatty acids DHA and EPA exhibited remarkable anticancer activities in MM cells (5, 22). MM cells showed greater sensitivity to DHA and EPA than other cells such as HT-29 colon cancer cells and A549 lung cancer cells, as well as primary human mast cells. DHA and EPA only slightly attenuated cell viability even at high concentrations (**Figure 1A**). These results suggested that these fatty acids might have selective cytotoxic potential on different human cells and prompted us to explore molecular mechanisms by which bortezomib and DHA/EPA induced cell death in MM.

Given the crucial role of necroptosis in the development of many types of human cancers (23), involvement of the necroptotic pathway was further investigated in bortezomib and DHA/EPA-induced cell death in MM cells. RNA-seq data showed that *HSP90* and *PUMA*, NF-κB target genes that have been shown to regulate the canonical necroptotic pathway (24, 25), and TNF receptor superfamily member 1A (*TNFRSF1A*) were upregulated in DHA/EPA or bortezomib-treated OPM2 cells (**Figure 1B**), suggesting that necroptosis may be involved. Furthermore, Kyoto Encyclopedia of Genes and Genomes (KEGG) pathway enrichment analysis indicated that necroptosis was indeed associated with DHA/EPA-induced cell death (adjusted P value _(DHAvsCtrl)_ = 0.01, adjusted P value _(EPAvsCtrl)_ = 0.004). In addition to OPM2 cell line, we have performed RNA-seq analysis in U266 cell line which was found to be resistant to both DHA and EPA (5). As shown in **Supplementary Figure 1**, KEGG pathway analysis showed that no cell death-related pathways were significantly enriched in DHA/EPA/bortezomib-treated cells in U266 cell line. Meanwhile, they showed almost no effect on the expression levels of HSP90, TNFRSF1A and PUMA, which suggests that NF-κB signaling pathway was not activated after treatment in U266 cells, indirectly supporting our hypothesis that NF-κB pathway-associated necroptosis might play a role in DHA/EPA/bortezomib-induced cell death in MM cells. Since L363, OPM2 and MM.1S cell lines are all sensitive to DHA and/or EPA, we only selected the OPM2 cell line for RNA-seq analysis.

MLKL oligomerization is a requisite step to execute necroptotic cell death (26). For this reason, the ability of MLKL to form oligomers in treated MM cells was evaluated. An irreversible chemical crosslinker bissulfosuccinimidyl suberate (BS^3^) was used to determine the oligomerization of MLKL. Upon TSZ stimulation in HT-29 cells, high molecular weight oligomers were readily observed in the presence of BS^3^ on nonreducing PAGE using antibodies against phospho-MLKL, C-terminus of MLKL and N-terminus of MLKL, respectively (**Figure S3**). In MM cells, it was demonstrated that both bortezomib and DHA induced the formation of MLKL oligomers (**Figure 1C**), suggesting that MLKL-dependent necroptosis was implicated in their cytotoxicity.

Because phosphorylation of human MLKL by RIPK3 is a key trigger for its oligomerization (27), it was subsequently investigated whether bortezomib and DHA/EPA induced phosphorylation of MLKL in MM cells. Surprisingly, phospho-MLKL was not detected in all MM cell lines at the expected position (~54 kDa). Instead, a smaller phosphorylated protein band (~35 kDa) was found in bortezomib and DHA/EPA-treated cells (**Figure 1D**). We have also checked the effect of these cell death triggers (DHA/EPA/bortezomib) on phosphorylation of MLKL in other MM cell lines. Only a faint increased band around 35KD was observed in RPMI8226 cell line (**Supplementary Figure 2**). In other MM cell lines, this band cannot be detected even with high amount of protein and after long-time exposure (not shown). This can be explained by the fact that the key upstream kinase of MLKL (RIPK3) was found to be absent in these MM cells (**Figure S4**), and, therefore, only small amount of C-terminal cleavage can be phosphorylated by the low abundant of RIPK3 in MM cells. The detection of the 35KD of phosphorylated-MLKL provides us a clue that there may exist an alternative mechanism of MLKL-dependent necroptosis in MM cells. The expression levels of necrosome components in other MM cells was tested in the next step. Strikingly, RIPK3, the key upstream kinase of MLKL, was found to be absent in almost all MM cell lines except for MM.1S (**Figure S4**), thereby explaining why no phosphorylated-MLKL (~54 kDa) could be detected in treated MM cells. Taken together, these results implied that there might be alternative mechanisms of MLKL-dependent necroptotic cell death in response to anticancer drugs in MM cells.

### Cell Death Triggers Reduce MLKL Levels in MM Cells

**Figure 2:**
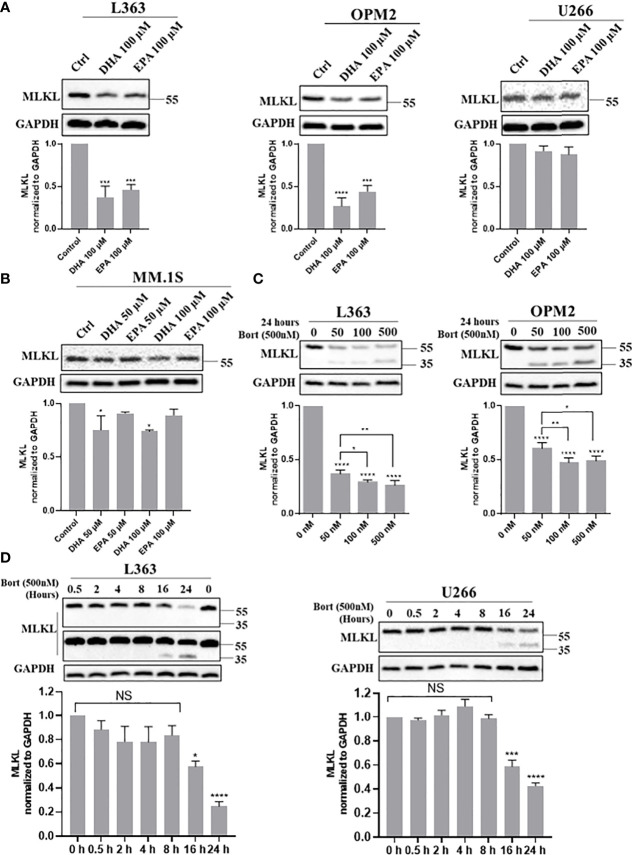
Cell death triggers down-regulate total MLKL and bortezomib induces a 35 kDa protein in MM cells. **(A, B)** L363, OPM2, U266 and MM.1S cells were treated with indicated concentrations of DHA/EPA for 24 h. **(C)** OPM2 and L363 cells were treated with indicated concentrations of bortezomib for 24 h. **(D)** L363 and U266 cells were treated with bortezomib (500 nM) for 0, 0.5, 2, 4, 8, 16 and 24 h. After treatments, cells were lysed with RIPA buffer and cell lysates were subjected to western blotting with antibodies against C-terminus and GAPDH. Data were expressed as mean ± SD of three independent experiments. *p < 0.05, **p < 0.01, ***p < 0.001, ****p < 0.0001 when compared with control, NS, no significance.

Since necroptosis-associated phosphorylation of MLKL was not observed, it was studied whether bortezomib and DHA/EPA altered MLKL protein levels in MM cells. Total MLKL was significantly decreased by the treatment of DHA/EPA for 24 h both in L363 and OPM2 cell lines, which have been previously demonstrated to be sensitive to DHA/EPA (**Figure 2A**, left and middle panel). In contrast, minor effects were found in a DHA/EPA-resistant cell line U266 (**Figure 2A**, right panel), while in the DHA-sensitive but EPA-resistant cell line MM.1S, only DHA treatment down-regulated total MLKL in a concentration-dependent manner (**Figure 2B**). The downregulation of total MLKL was also observed in TS- (apoptotic condition) or TSZ- (necroptotic condition) treated MM cells (**Figure S5**), indicating alternative mechanisms of MLKL-dependent necroptotic cell death in MM cells. In addition, bortezomib caused a potent decrease of MLKL in all MM cell lines in a concentration- and time-dependent manner, as shown in **Figures 2C, D**. Interestingly, a truncated protein of approximately 35 kDa was detected upon bortezomib treatment as detected by an antibody that specifically recognizes the C-terminus (**Figures 2C, D**). This 35 kDa protein was present after 16 h treatment (**Figure 2D**), a time point at which the cytotoxicity of bortezomib developed (**Figure S6**).

Given that the 35 kDa protein was only detected in the presence of proteasome inhibitor bortezomib (**Figure S7**), it was investigated whether another proteasome inhibitor, such as MG132, induced the 35 kDa protein in MM cells as well. Similar to bortezomib, a pronounced decrease of total MLKL accompanied by the appearance of the 35 kDa protein was observed in MG132-treated cells also (**Figure 4F**, lanes 2 and 6-8). Based on these findings, it was hypothesized that MLKL may undergo cleavage in response to death triggers, such as bortezomib, MG132 or DHA/EPA in MM cells and the resultant C-terminal fragment (~35 kDa) maybe quickly be degraded by the ubiquitin–proteasome system.

### MLKL Is Indeed Cleaved by the Induction of Bortezomib in MM Cells

**Figure 3:**
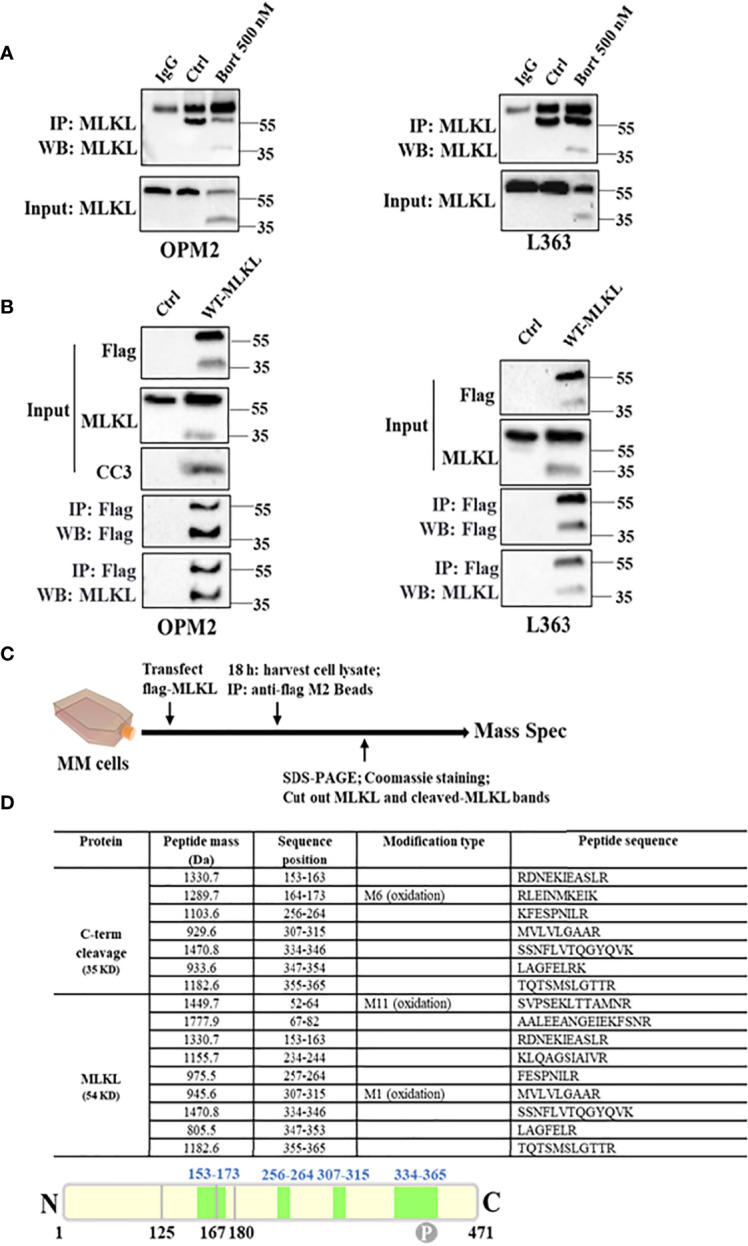
MLKL is indeed cleaved upon cell death induction in MM cells. **(A)** Endogenous MLKL immunoprecipitates prepared from DMSO or bortezomib-treated OPM2 and L363 cells were analyzed by western blotting with antibody for C-terminus. Protein G Dynabeads with lysis buffer was used as negative control and the detected band around 55 kDa was the IgG heavy chain. The input lysates were also analyzed by western blotting. **(B)** OPM2 and L363 cells were transiently transfected with 5 µg C-terminal Flag-MLKL vector encoding wild-type MLKL. After 18 h, exogenous MLKL was immunoprecipitated from transfected or non-transfected cells with anti-Flag M2 beads. The input lysates and immunoprecipitates were analyzed by western blotting with indicated antibodies. **(C)** Outline of MLKL cleavage study experiment. Exogenous MLKL immunoprecipitates obtained as described in **(B)** were subjected to reducing SDS-PAGE and proteins were stained with Coomassie stain. Then, total MLKL and C-terminus bands were excised and performed for mass spectrometric analysis. **(D)** Identified Peptides from total MLKL and C-terminus by mass spectrometric analysis. M, the specific amino acid was modified by oxidation.

To directly validate the potential cleavage of MLKL, endogenous MLKL was pulled down from DMSO or bortezomib-treated OPM2 and L363 cells using an anti-C-terminal MLKL antibody. Immunoblotting with the same antibody demonstrated that both the total MLKL and its C-terminal fragment were present in the pull-down samples from bortezomib-treated cells and only total MLKL was detected in DMSO-treated cells (**Figure 3A**). Furthermore, we overexpressed wild-type MLKL-Flag (MLKL^WT^-Flag) in OPM2 and L363 cells and immunoprecipitation of MLKL was preformed using anti-Flag beads. As shown in **Figure 3B**, overexpression of MLKL itself already showed strong toxicity in MM cells due to activation of caspase-3 after transfection, which is consistent with earlier observations (28). Expectedly, without any additional treatment, overexpression of MLKL alone sufficed to induce the appearance of the C-terminal fragment, while the exogenous C-terminal fragment was detected in the immunoprecipitated proteins of wild-type MLKL-expressing cells with the anti-C-terminal MLKL antibody.

Taking into account the size of the C-terminal fragment and the pivotal role of the N-terminal domain in necroptosis execution, it was speculated that the cleavage site should be located within the first α-helix of the BR domain of MLKL (126-167 aa). To further determine the cleavage site of MLKL, mass spectrometric analysis on trypsin-digested exogenous C-terminal fragment was performed (**Figure 3C**). Mass spectrometric analysis identified 7 peptides from the C-terminal fragment (**Figure 3D**), thereby confirming that the 35 kDa fragment was derived from human MLKL. A unique peptide with sequence of _153_RDNEKIEASLR_163_ was identified from the C-Flag-MLKL (**Figure 3D**), indicating that the cleavage site was presumably located between 126 aa and 153 aa.

### Activated Caspases Catalyze MLKL Cleavage in MM Cells

**Figure 4:**
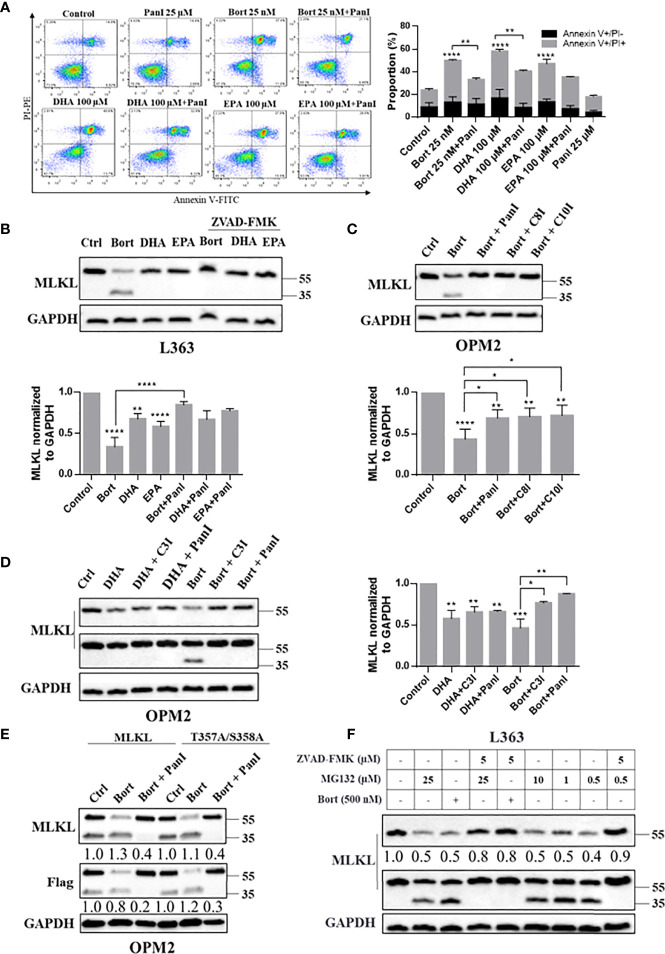
Caspase-3/8/10 cleave MLKL in MM cells after cell death induction. **(A)** OPM2 cells were preincubated with or without Pan-caspase inhibitor ZVAD-FMK (20 µM) for 1 h and were then treated with bortezomib (25 nM) or DHA/EPA (50 µM) for 24 h. Cell death was determined by Annexin V and PI staining. **(B)** L363 cells were preincubated with or without pan-caspase inhibitor ZVAD-FMK (5 µM) for 1 h and were then treated with the indicated compounds for 24 h. **(C)** OPM2 cells were preincubated with or without indicated caspase inhibitors ZVAD-FMK, ZIETD-FMK, ZAEVD-FMK **(D)** or Z-DEVD-FMK (5 µM) for 1 h and were then treated with bortezomib (50 nM) or DHA (50 µM) for 24 h. **(E)** OPM2 cells were transfected with 5 µg of vectors encoding C-terminal Flag-MLKL^WT^ or C-terminal Flag-MLKL^T357A/S358A^. After 18 h, transfected cells were pretreated with or without ZVAD-FMK (5 µM) for 1 h before treatment with bortezomib (50 nM) for 24 h. **(F)** L363 cells were treated with 500 nM of bortezomib or indicated concentrations of MG132 for 24 h in the absent or present of 1 h preincubation with ZVAD-FMK (5 µM). After treatments, whole cell lysates were immunoblotted with indicated antibodies. Data were expressed as mean ± SD of. *p < 0.05, **p < 0.01, ***p < 0.001, ****p<0.0001 when compared to control. The fold changes of C-MLKL, C-Flag or total MLKL in the respective lanes in **(E, F)** were normalized to GAPDH and quantification numbers are presented in the bottom of corresponding proteins.

The above findings raised questions about the executors of MLKL cleavage and the exact location of the potential cleavage site. We found that pan-caspase inhibitor ZVAD-FMK efficiently prevented DHA/EPA and bortezomib induced cell death in OPM2 cells (**Figure 4A**), which is consistent with our earlier findings in primary cells from MM patients (22). Thus, we next examined whether MLKL was cleaved by caspases (29) in MM cells. Western blot analysis showed that bortezomib and DHA/EPA-induced MLKL down-regulation was almost completely blocked by the addition of pan-caspase inhibitor ZVAD-FMK, while the generation of the C-terminal MLKL fragment was markedly reduced (**Figure 4B**). Bortezomib and DHA/EPA have been previously found to be able to activate caspase-3/8 in MM cells (30). Additionally, caspase-10 activity has been demonstrated to be required for MM cell survival (31). We therefore investigated whether MLKL was a direct substrate of caspase-3/8/10. As shown in **Figures 4C**, **S6A**, caspase-8 inhibitor Z-IETD-FMK and caspase-10 inhibitor Z-AEVD-FMK were found to be able to block bortezomib-induced MLKL cleavage in OPM2 and L363 cells, respectively. Identical results were also obtained by treating cells with caspase-3 inhibitor Z-DEVD-FMK (**Figure 4D**).

**Figure 5:**
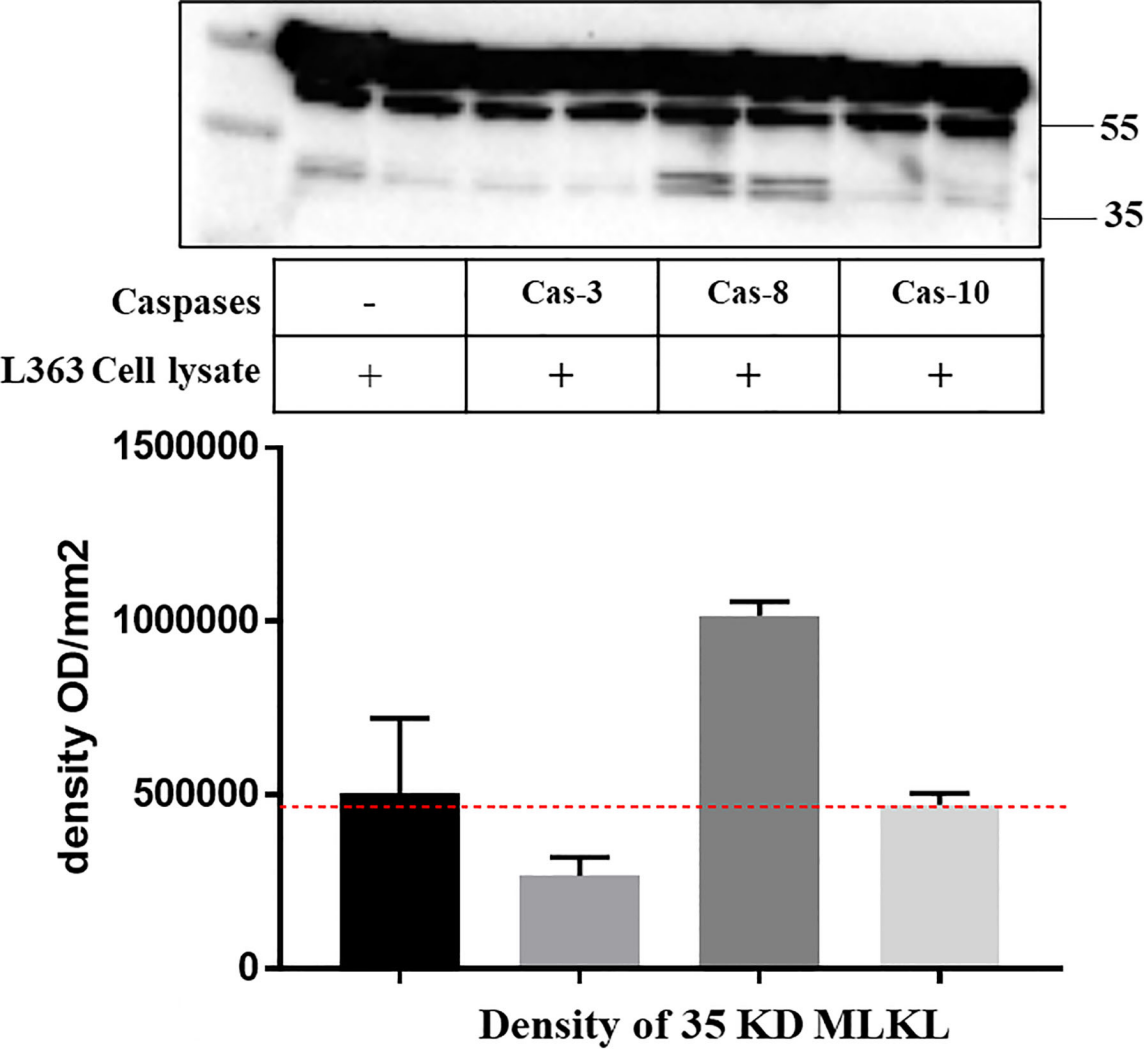
Recombinant caspase-8 induces endogenous MLKL cleavage in L363 cell line. Cell lysate of L363 cell line was incubated with or without indicated recombinant caspases (2 U) at 37°C for 1 h. Whole cell lysates were analyzed by western blotting with an antibody that specifically recognizes the C-terminal of MLKL. The image signal intensity of the C-terminal MLKL cleavage was presented below the blot image.

Consistent with the results above, the pan-caspase inhibitor ZVAD-FMK substantially reduced the level of bortezomib-induced C-terminal MLKL fragment in MLKL^WT^-Flag expressing MM cells (**Figure 4E** (lanes 1-3) and S8B). We further examined whether phosphorylation site mutant MLKL-Flag (MLKL^T357A/S358A^-Flag) can be cleaved by caspases in MM cells. Interestingly, it is notable that the C-terminal fragment of Flag-MLKL can also be detected in MLKL^T357A/S358A^-Flag expressing cells (**Figure S7**), indicating that a RIPK3-dependent phosphorylation (Thr357/Ser358) was not needed for MLKL cleavage in MM cells. In line with previous results, we found that the pan-caspase inhibitor almost entirely blocked the bortezomib-induced cleavage of MLKL^T357A/S358A^-Flag (**Figure 4E**, lanes 4-6). Moreover, increases in the C-terminal MLKL fragment triggered by proteasome inhibitor MG132 in L363 cells was also inhibited by pan-caspase inhibitor (**Figure 4F**, lanes 4 and 9). Also endogenous MLKL from MM cells was found to be cleaved by recombinant caspase-8 (**Figure 5**). Together, this indicates that MLKL is a novel substrate of caspase-3/8/10 in MM cells.

### MLKL Is Cleaved at Asp140 Upon Cell Death Induction in MM Cells

**Figure 6:**
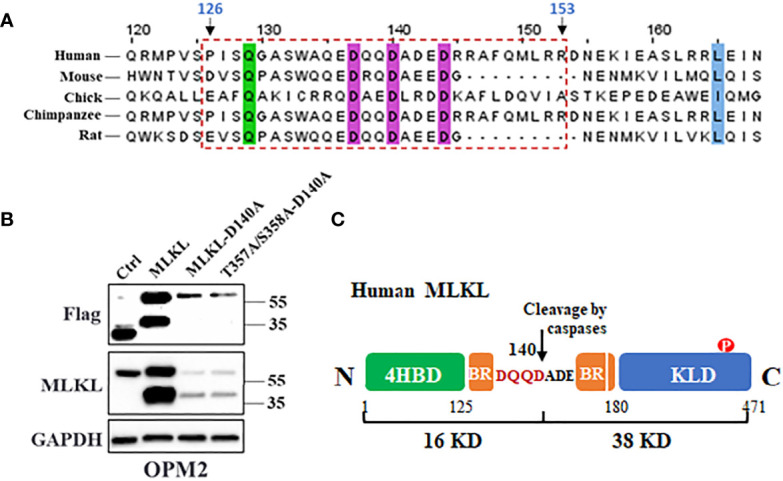
MLKL is cleaved at Asp140 upon cell death induction in MM cells. **(A)** Amino acid sequences alignment of the first α-helix of MLKL BR domain from multiple species. The conserved residues are highlighted with colored shading and the potential cleavage region (126-153 aa) was indicated in a rectangle with dashed border. **(B)** OPM2 cells were transfected with 5 µg of vectors encoding C-terminal Flag-MLKL^WT^, C-terminal Flag-MLKL^D140A^ or C-terminal Flag-MLKL^T357A/S358A-D140A^. After 18 h, whole cell lysates were subjected to western blotting with antibodies for C-terminus, Flag and GAPDH. **(C)** Cartoon diagram of MLKL structure and the cleavage by caspases. Human MLKL can be cleaved by activated caspases at Asp140 in the BR that produce a 16 kDa (N-terminus) and a 38 kDa (C-terminus) fragments.

Apoptotic caspases including caspase-3/8/10 cleave their substrates following a motif as DXXD-A/G/S/T (32, 33). Alignment of primary sequences of the first α-helix of MLKL BR domain from multiple species predicted that Asp140 could be the potential caspase cleavage site of human MLKL, which would produce a C-terminal 38 kDa fragment (**Figure 6A**). To test this hypothesis, Asp140 of MLKL^WT^-Flag and MLKL^T357A/S358A^-Flag were respectively substituted with an alanine to generate loss-of-function mutants as MLKL^D140A^-Flag and MLKL^T357A/S358A-D140A^-Flag. As shown in **Figure 6B**, the C-terminal MLKL fragments were not visible in western blot analysis of samples from MLKL^D140A^-Flag and MLKL^T357A/S358A-D140A^-Flag expressing MM cells using anti-Flag antibody, suggesting that Asp140 was involved in the caspase-mediated cleavage of MLKL after cell death induction in MM cells (**Figure 6C**).

### DHA, EPA and Bortezomib Trigger the Induction of Nuclear Translocation of Total MLKL and C-Terminal MLKL in MM Cells

**Figure 7:**
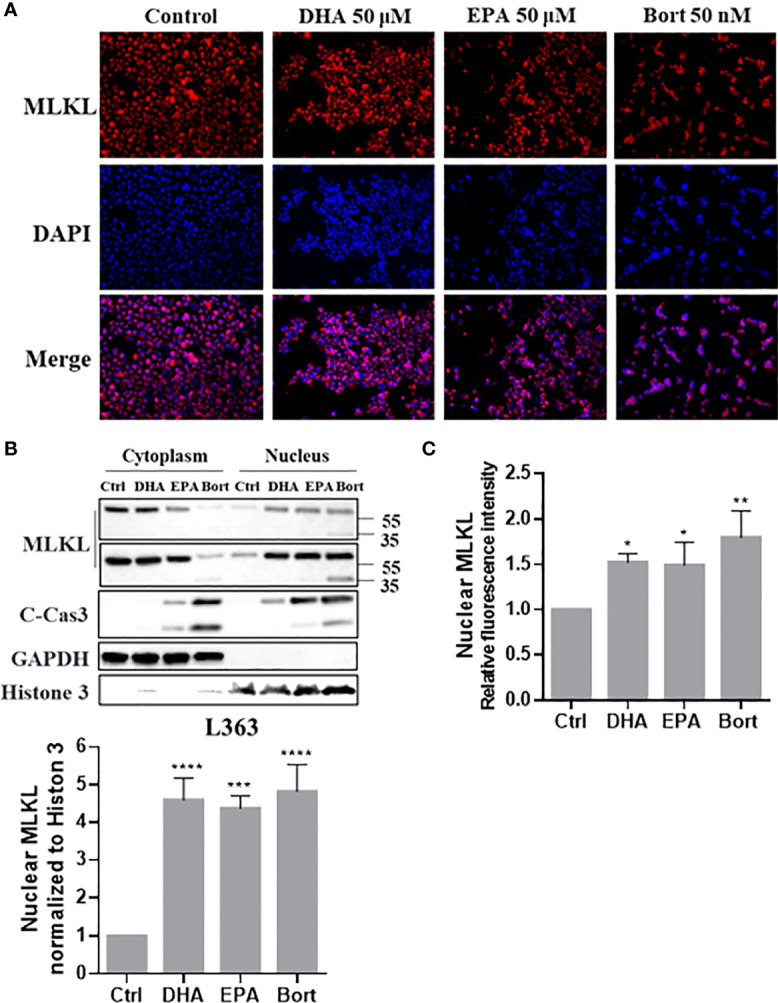
Cell death triggers induce nuclear translocation of total MLKL and C-erminal MLKL in MM cells. L363 cells were treated with 50 µM of DHA/EPA or 50 nM bortezomib for 24 h. After treatment, **(A)** cells were stained with primary anti-MLKL antibody and AlexaFluor 594-conjugated secondary antibody (red) and the nucleus was stained with DAPI (blue). Then, stained cells were examined using a Fluorescence microscopy using specific fluorescence channel with 20x objective. The red MLKL and blue nucleus were merged to purple fluorescence using Image J software. **(B)** Cytoplasmic and nuclear extracts were analyzed by western blotting using the indicated antibodies. **(C)** Quantification of **(A)** using Image J software. Data were expressed as mean ± SD of three independent experiments. *p < 0.05, **p < 0.01, ***p < 0.001, ****p < 0.0001 when compared with control.

It was previously shown that phosphorylated MLKL can translocate into the nucleus and contribute to cell death during TSZ-induced necroptosis. To investigate if this occurred during necroptosis of RIPK3-deficient MM cells, cellular distribution of MLKL was further analyzed. Interestingly, MLKL significantly accumulated in nucleus of MM cells treated with DHA, EPA and bortezomib (**Figure 7B**) and MLKL nuclear translocation was also evidenced by immunocytochemistry analysis (**Figures 7A, C**). The bortezomib-induced C-terminal fragment of MLKL was also present in nucleus (**Figure 7B**). These results indicated that cell death triggers not only induce total MLKL cleavage, but also nuclear translocation of the C-terminal fragment in MM cells.

## Discussion

**Figure 8:**
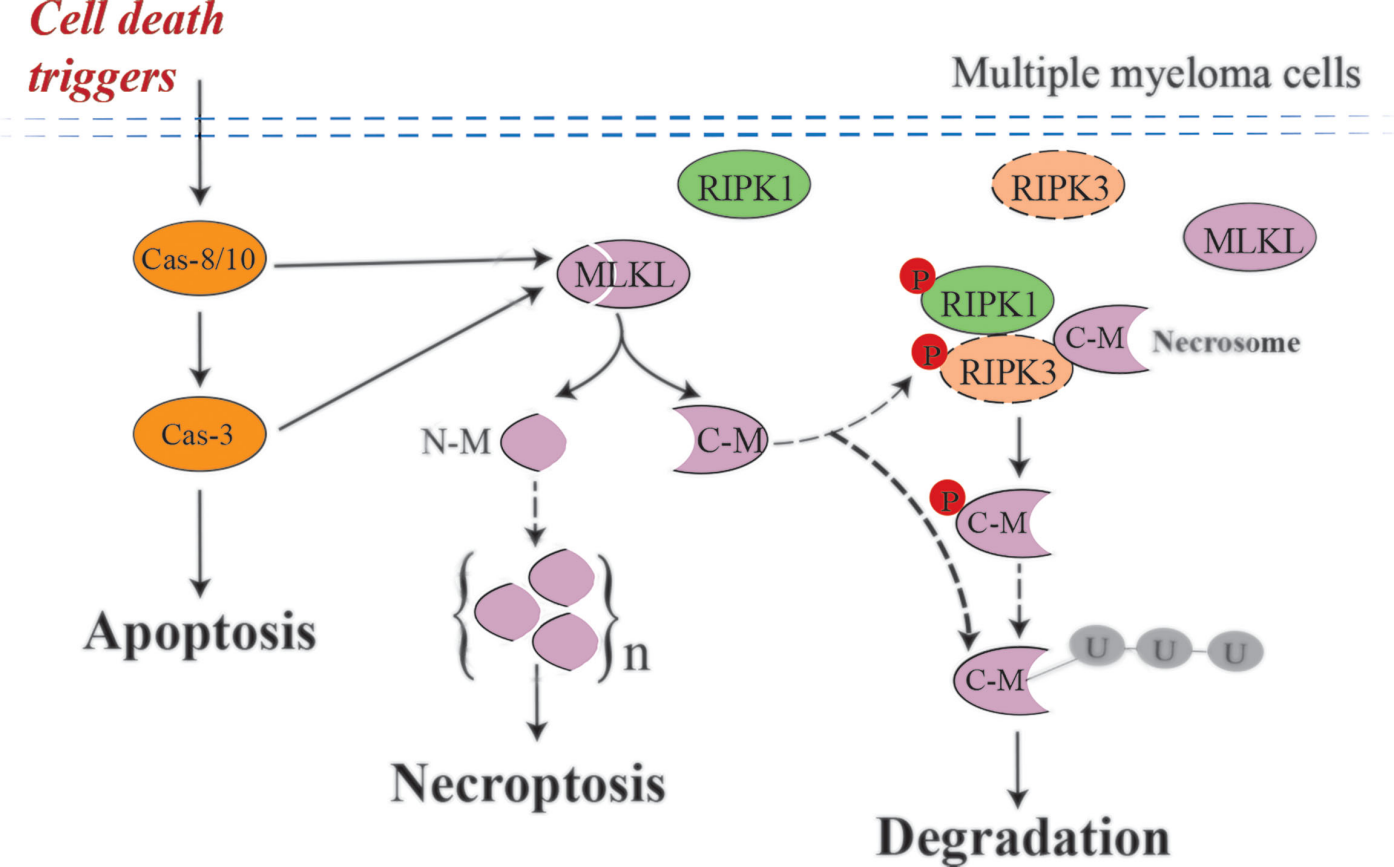
Possible mechanism of MLKL cleavage-mediated necroptotic activation in MM cells. Cell death triggers, such as DHA, EPA and bortezomib, induce activated caspases-mediated MLKL cleavage in multiple myeloma cells, which are RIPK3-deficient cells. After cleavage, the N-terminal cleavage induce necroptosis by oligomerization and the C-terminal cleavage may be degraded by the ubiquitin–proteasome system. Small amounts of C-terminal cleavage can be phosphorylated by low abundant RIPK3 existing in MM cells and subsequently degraded. Dashed arrows, need to be further investigated.

In the present study, we show that cell death triggers such as DHA, EPA and bortezomib may induce necroptosis in multiple myeloma cells independent of RIPK3. We further demonstrated that this process is accompanied by MLKL cleavage mediated by activated caspases (**Figure 8**). To date, the image of canonical necroptotic pathway has been well lined out: induction of necroptosis by e.g., TSZ leads to phosphorylation of MLKL by RIPK3. Phosphorylated MLKL subsequently undergoes a conformational change that exposes its N-terminal domain (NTD), triggering its oligomerization to octamers and membrane permeabilization (34). Our findings opened new insights into how MLKL may drive necroptosis in RIPK3-deficient cells.

### MLKL Is Cleaved at Asp140 in MM Cells After Cell Death Induction

Our study shows for the first time that MLKL can be cleaved at Asp140 into two unique fragments (i.e., a 16 kDa N-terminus and a 38 kDa C-terminus fragment). Recent studies demonstrated that the NTD truncation of MLKL is sufficient to induce necroptosis in HEK293T cells and mouse dermal fibroblasts (13, 14, 35), suggesting that the NTD domain of MLKL may be the necroptotic death effector domain and the C-terminal KLD domain of MLKL will act as a suppressor of MLKL-mediated necroptosis. Notably, the NTD domain with one α-helix of BR (1-167 aa) is unable to induce necroptosis (13), possibly because the first α-helix of BR (126-167 aa) still blocked the exposure of the death effector domain to form oligomers. Our results show that bortezomib and DHA induced MLKL oligomerization in MM cells, indicating their capabilities in activating necroptosis. Importantly, the 1-140 aa of human MLKL was demonstrated to be able to form oligomers and induce necroptosis (36). This “indirect” evidence strongly suggests that the N-terminus oligomerizes with itself and move to plasma membrane, where it exerts its pore-forming function. Additionally, in light of our finding that only proteasome inhibitors induced the appearance of the C-terminal fragment of MLKL, we speculate that the C-terminus may be degraded shortly after cleavage by the ubiquitin-proteasome system. Notably, MLKL inhibitor BI-8925 was found to be able to block MLKL cleavage in MM cells (**Figure S10**), indicating that the Cys86 in the NTD domain may be critical for MLKL cleavage. Furthermore, since these anti-myeloma agents exhibited potent anticancer effects against MM cells from patients (22), it is reasonable to assume that caspase-mediated MLKL cleavage also occurs in primary MM cells undergoing necroptotic cell death. Future experiments are needed to confirm this assumption.

### RIPK3-Dependent Phosphorylation of MLKL in Not Required for MLKL Cleavage

RIPK3-dependent phosphorylation of MLKL at Thr357/Ser358 is regarded as a biomarker of necroptosis induction (6). Surprisingly, RIPK3 was not detectable in several MM cell lines, while also phosphorylated MLKL was not found after treatment with cytotoxic agents. This pointed to other RIPK3-independent mechanisms driving necroptosis in MM cells. Mutations of the phosphorylation sites (T357A/S358A) were apparently unable to prevent the cleavage of MLKL, suggesting that RIPK3-dependent phosphorylation is not required for MLKL cleavage and subsequent N-terminal cleavage-induced necroptosis.

### Cell Death Triggers Induce RIPK1 Cleavage in MM Cells

RIPK1 is a key upstream kinase of RIPK3 during TSZ-induced canonical necroptosis. In this study, we therefore examined whether these cell death triggers affected RIPK1 activity in MM cells. Phosphorylation of RIPK1 at Ser166 has been regarded as a biomarker for RIPK1 activation (37), but this was not detected in our study. Notably, we found that bortezomib and DHA/EPA induced RIPK1 cleavage in MM cells (**Figure S11**). Previous studies have reported that activated caspase-8 cleaves human RIPK1 at Asp324 to produce a 37 kDa (N-terminus) and a 39 kDa (C-terminus) fragments, resulting in blocking of NF-κB activation and promoting apoptosis in TNFα-induced apoptotic cells (38). Also, caspase-8-dependent cleavage of RIPK1 was found to suppress canonical necroptosis through disrupting the necrosome formation (7). Our observations of bortezomib and DHA/EPA-induced RIPK1 cleavage and RIPK1 activity-independent necroptosis in MM cells may suggest that RIPK1 cleavage could contribute to apoptosis after cell death induction. In addition, we also observed RIPK3 cleavage in RIPK3-expressing cell line MM.1S after cell death induction and there was no detectable phosphorylated RIPK3 (**Figure S11**), similar to our data of RIPK1. Further experimental work is needed to substantiate the cellular implications of the cleavage of these kinases in MM cells.

### Caspases-3/8/10 Cleave MLKL in MM Cells

We further addressed how MLKL is cleaved after treatment of MM cells with cytotoxic agents. Caspases are best-known intracellular proteases that hydrolyze a broad range of protein substrates by recognizing specific cleavage motifs in their sequences. Previous studies have shown that these anti-myeloma agents induced apoptosis through activating caspase-3 and -8 in MM cells (5), while caspase-10 was shown to play pivotal roles in maintaining MM cell survival by balancing the pro-survival and pro-death effects of autophagy (31). We demonstrate that specific inhibitors of these caspase-3, -8 and -10 and the pan-caspase inhibitor ZVAD-FMK reduced the cleavage of MLKL in bortezomib-treated MM cells (**Figures 4B–D**) and endogenous MLKL from MM cells was found to be cleaved by recombinant caspase-8 (**Figure 5**), indicating the involvement of caspases in the cleavage of MLKL. Moreover, it was demonstrated that ZVAD-FMK greatly inhibited bortezomib- and DHA/EPA-induced cell death in OPM2 cells (**Figure 4A**), indicating the crucial importance of caspase activation in inducing cell death by these agents. In addition to apoptotic caspases, inflammatory caspases are also associated closely with intracellular signal transduction (39). A recent study showed that MLKL pore formation activated the NLRP3 inflammasome, which resulted into the cleavage of inflammatory caspase-1 and activation of IL-1β (40). Moreover, elevated expression of caspase-1 was found in patient-derived MM cells (41). Since bortezomib and DHA induced MLKL oligomerization, it could be speculated that also inflammatory caspases may be involved in the cleavage of MLKL in MM cells.

### Caspase-8 Activity May Not Be Essential for N-Terminus -Induced Necroptosis

Caspase-8 activation is a biomarker for extrinsic (death receptor) apoptotic pathway (42) and blocking caspase-8 activity is required for initiating the canonical necroptotic pathway. This raised another inevitable question: why MLKL can induce necroptosis without caspase-8 inhibition in MM cells? Several studies have demonstrated that the NTD (1-125 aa) was required and sufficient to induce its oligomerization and trigger necroptosis. Importantly, a recent study showed that the 1-140 aa of human MLKL was able to form oligomers and induce necroptosis. Moreover, during the canonical necroptosis, phosphorylated-MLKL undergoes conformational changes, which results in release of the NTD and BR, allowing the formation of oligomers and subsequent plasma membrane rupture. Thus, this suggested that the C-terminal KLD of MLKL mainly acts as a suppressor of MLKL activity under normal conditions and does not seem to be essential for executing canonical necroptosis. We have previously reported that fatty acids DHA and EPA triggered cell death in patient-derived primary MM cells in a partly caspase-dependent manner (22), which supports our result that blocking caspase activity using pan-caspase inhibitor cannot completely inhibit cell death in MM cells. Blocking caspase-8 activity can inhibit apoptosis but induces necrosome formation when necrosome components are highly expressed (43). Under such conditions, cell death triggers induce either apoptosis or necroptosis. However, we observed that bortezomib and DHA/EPA induce necroptosis *via* caspase-mediated MLKL cleavage in MM cells. It is conceivable that under these conditions induction of necroptosis may be independent of necrosome formation. Interestingly, a recent study showed that proteasome inhibitors bortezomib and MG132 activated the canonical necroptotic pathway in RIPK3-expressing mouse fibroblasts and human leukemia cells without caspase-8 inhibition (44), confirming that inhibition of caspase-8 activity may not be essential for induction of necroptosis. The role of MLKL cleavage in cell death triggered by bortezomib and DHA and EPA, should be further investigated in MM cells deficient in MLKL. Unfortunately, siRNA transfection to knockdown MLKL was found to be highly toxic to MM cells, which resulted in caspase activation and subsequent MLKL cleavage, ultimately leading to cell death (data not shown). Further research is needed to delineate the role of MLKL cleavage in this cell death pathway.

### After Cleavage, Total MLKL and C-Terminus Translocate Into Nucleus

Phosphorylated MLKL can translocate into nucleus along with RIPK1 and RIPK3 to contribute to necrosome formation and necroptosis (15, 16). This nuclear translocation of MLKL might be initiated by its phosphorylation-induced conformational change that exposes the C-terminal nuclear localization signal (NLS, 224-256 aa) during necroptosis. Our study showed that the anti-myeloma agents induce not only nuclear translocation of total MLKL but also of the C-terminal fragment in MM cells, suggesting that the NLS motif in the C-terminal fragment was exposed after cleavage. This observation leads to another two questions: why non-phosphorylated MLKL in MM cells can translocate into nucleus and what is the consequence of the nuclear translocation of the C-terminal fragment? Based on our finding of caspases-mediated MLKL cleavage, it is reasonable to assume that the interaction between activated caspases and MLKL may cause conformational changes in MLKL to expose the C-terminal NLS motif, which allows import of MLKL into the nucleus. Moreover, it is well known that executioner caspases cleave many nuclear substrates to create nuclear morphological changes during apoptosis (45). Our data also showed that DHA/EPA and bortezomib markedly increased nuclear cleaved-caspase3 (**Figure 7B**). Since there is no NLS motif in their sequences, translocation is possible facilitated by carrier proteins (e.g., AKAP95) (46–48). Hence, we hypothesize that when MLKL binds to activated caspases after cell death induction, these caspases might be imported into the nucleus together with MLKL to facilitate apoptosis. Further investigation is ongoing to confirm this assumption.

## Conclusion

Necroptosis has been implicated in a wide range of human diseases. A better understanding of the underlying regulatory mechanisms would help development of new therapeutics for necroptosis-associated human diseases. The present study revealed a novel regulatory mechanism for necroptosis in RIPK3-deficient MM cells: cell death triggers induce caspase-mediated MLKL cleavage to activate MLKL to induce necroptosis. There are still many unsolved questions: does MLKL cleavage occur in other RIPK3-deficient cancer cells and is the C-terminal cleavage ubiquitinated and degraded by proteasome after cleavage. Also, how do the N-terminal fragments form oligomers and what is the exact consequence of the nuclear translocation of the C-terminal fragment. The novel regulatory mechanisms of necroptosis found are not paradoxical with the canonical necroptosis: in RIPK3-expressing cancer cells, the necrosome-mediated canonical necroptotic pathway is activated through phosphorylation of MLKL, whereas in RIPK3-deficient cancer cells caspase-mediated MLKL cleavage may be the trigger to induce the cell death induction (**Figure 8**). Fatty acids DHA and EPA have been documented to induce ferroptosis in multiple cancer cells, such as leukemia and head and neck carcinoma (49, 50). Therefore, other cell death pathways such as ferroptosis and pyroptosis (51) may also contribute to DHA/EPA-induced cell death in MM cells and the importance of these pathways should be further investigated in future studies. Taken together, the present study suggests a novel role and regulatory mechanisms of necroptosis in multiple myeloma, and may provide a new molecular target (avenue) for therapeutic intervention of certain human cancers with eliminated RIPK3 function.

## Data Availability Statement

The RNAseq data presented in the study are deposited in the ArrayExpress repository and will be available under accession number E-MTAB-11907.

## Author Contributions

FR initiated and supervised the study. JC and FR designed the study and wrote the manuscript. JC, BB and SW performed experiments and analyzed data. FR, RR and JG guided the study and critically revised the manuscript. All authors read and approved the final manuscript.

## Funding

This work was financially supported by Nutricia Research and the Chinese Scholarship Council (CSC) (File No.201506180025). The funders had no role in the study design, data collection and analysis and preparation of the manuscript.

## Conflict of Interest

Author RR is currently employed by company Genmab B.V.

The remaining authors declare that the research was conducted in the absence of any commercial or financial relationships that could be construed as a potential conflict of interest.

## Publisher’s Note

All claims expressed in this article are solely those of the authors and do not necessarily represent those of their affiliated organizations, or those of the publisher, the editors and the reviewers. Any product that may be evaluated in this article, or claim that may be made by its manufacturer, is not guaranteed or endorsed by the publisher.
