# Supplementary material for: Cell Death Triggers Induce MLKL Cleavage in Multiple Myeloma Cells, Which may Promote Cell Death
Source: Front Oncol. 2022 Jul 28;12:907036. doi: 10.3389/fonc.2022.907036 (PMC9369655; doi:10.3389/fonc.2022.907036)
Supplement: Supplementary file 1 [file DataSheet_1.docx]

Supplementary Material

# Supplementary Figures


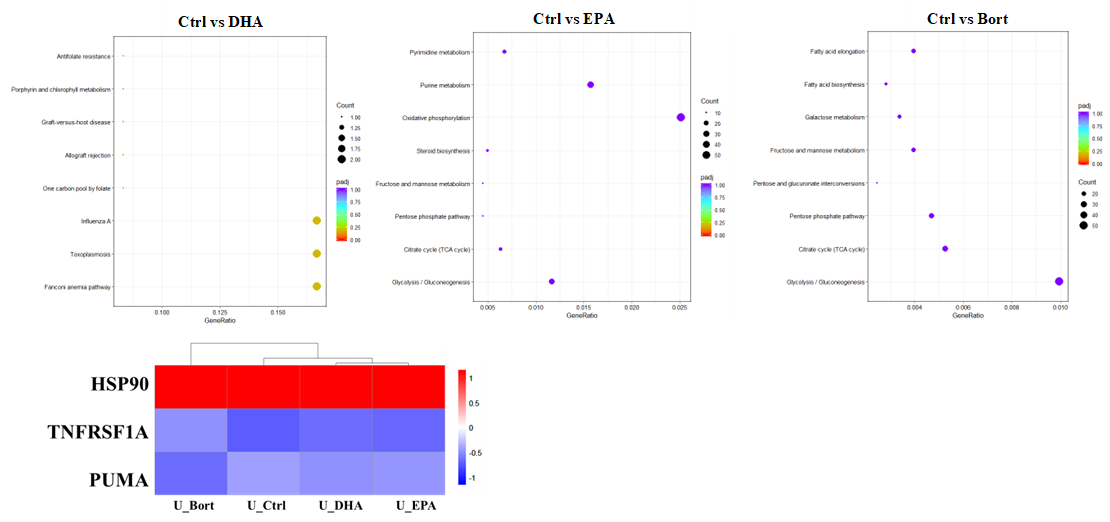


B

A

**Supplementary Figure 1** (A) KEGG pathway analysis of DEGs in DHA/EPA or bortezomib treated U266 cells compared to control. The counts present the number of DEGs enriched in a particular pathway. Different colors represent Padj values. (B) Heatmaps depicting the expression levels of HSP90, TNFRSF1A and PUMA in treated U266 cells.


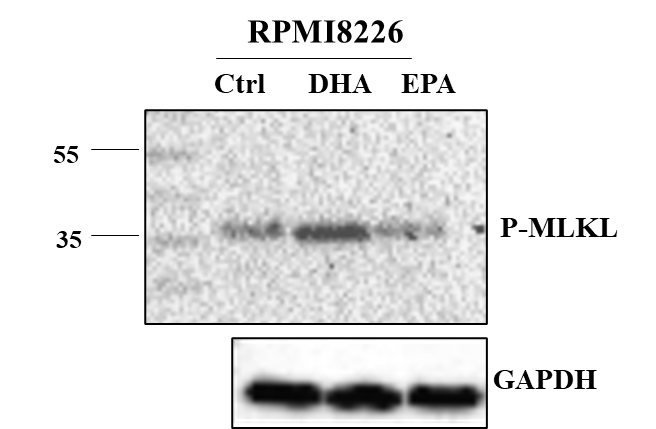


**
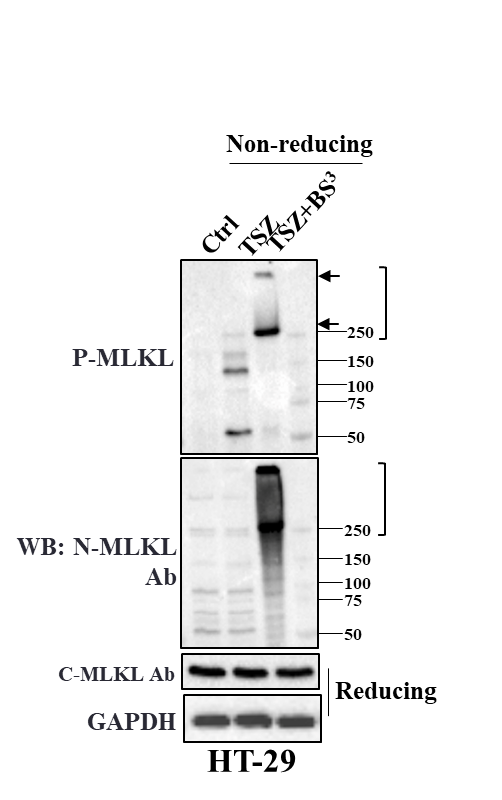
Supplementary Figure 2** The effect of DHA/EPA on phosphorylation of MLKL in RPMI8226 cell line. RPMI8226 cells were treated with 100 µM of DHA or EPA and 500 nM of bortezomib for 24 h and lysed with RIPA buffer. Whole cell lysates were subjected to western blotting with antibodies against p-MLKL and GAPDH.

**Supplementary Figure 3.** Necroptotic condition induce phosphorylated MLKL oligomerization in HT-29 cells. HT-29 cells were treated with DMSO or necroptotic conditions [TNFα (30 ng/ml), SM-164 (10 nM) and ZVAD-FMK (20 µM)] for 5 h. Cells were lysed with lysis buffer after 1 h incubation with or without crosslinker BS3 (0.5 mM). Then, cell lysates were resolved on non-reducing and reducing SDS-PAGE and immunoblotted with the antibodies against p-MLKL, C-terminal of MLKL, N-terminal of MLKL and GAPDH.


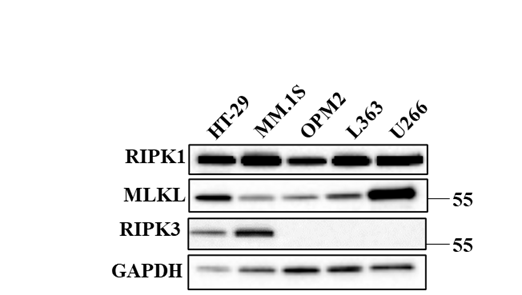


**Supplementary Figure 4.** The expression levels of necrosome components in MM cell lines. HT-29, OPM2, L363, U266 and MM.1S cells were lysed with RIPA buffer and whole cell lysates were analyzed by western blotting with indicated antibodies.


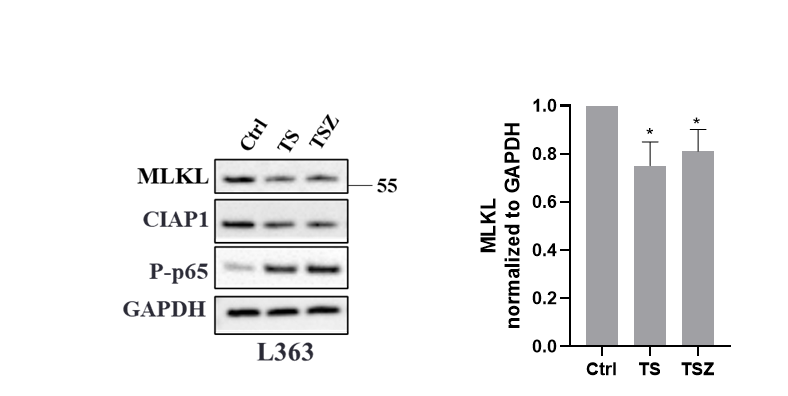


**Supplementary Figure 5.** The effect of typical apoptosis or necroptosis induction in L363 cells. Cells were treated with apoptotic condition [TNFα (30 ng/ml) and SM-164 (10 nM)] (TS) or necroptotic condition [TNFα (30 ng/ml), SM-164 (10 nM) and ZVAD-FMK (20 µM)] (TSZ) for 24 h. Whole cell lysates were analyzed by western blotting with antibodies for CIAP1, P-p65, C-terminal of MLKL and GAPDH. Data were expressed as mean ± SD (**p* < 0.05).


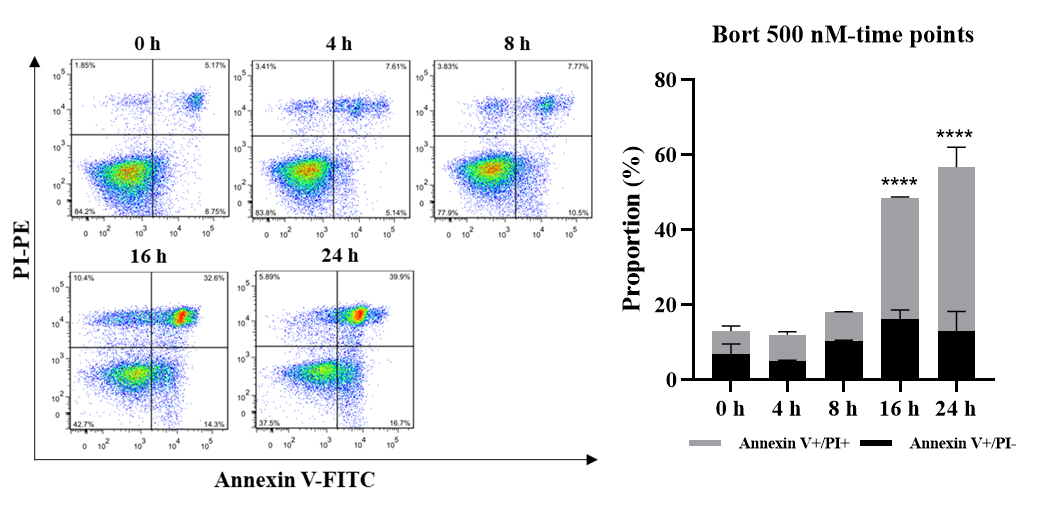


**Supplementary Figure 6.** The effect of bortezomib at different time points on cell death in MM cells. Cells were treated with 500 nM bortezomib for 0, 4, 8, 16 and 24 h. Then cell death was determined by Annexin-V and PI staining. Data are presented as mean ± SD of three independent repeats. *****p* < 0.0001when compared with 0 h.

**
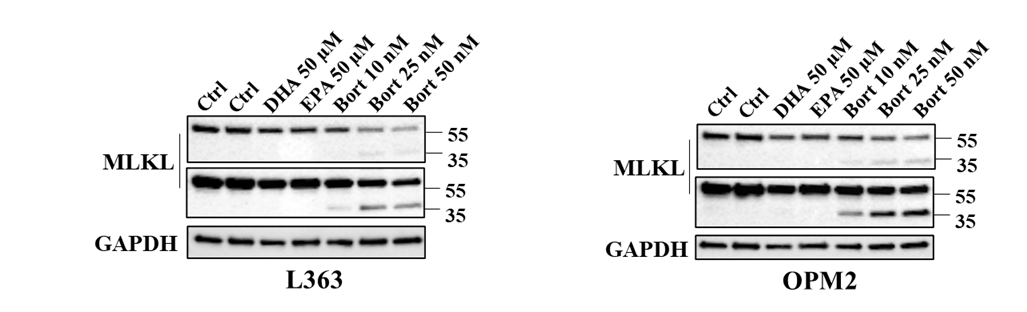
**

**Supplementary Figure 7.** Only bortezomib induce the appearance of the 35 kDa protein in MM cells but not DHA and EPA. L363 and OPM2 cells were treated with 50 µM of DHA or EPA or indicated concentrations of bortezomib for 24 h and lysed with RIPA buffer for western blotting with antibodies against C-terminal of MLKL and GAPDH.


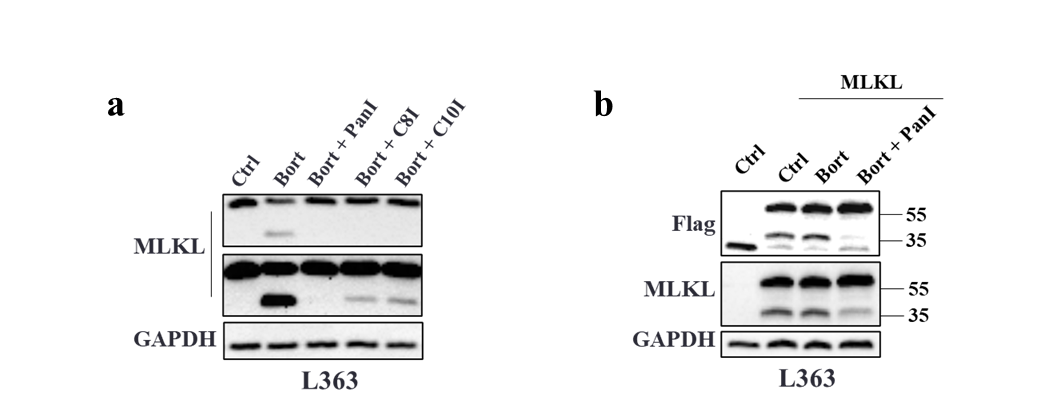


**Supplementary Figure 8.**  Caspase inhibitors block MLKL cleavage in L363 cell line after cell death induction. (a) L363 cells were preincubated with or without indicated caspase inhibitors ZVAD-FMK, ZIETD-FMK or ZAEVD-FMK (5 µM) for 1 h and were then treated with bortezomib (50 nM) for 24 h. (b) L363 cells were transfected with 5 µg of vectors encoding C-terminal Flag-MLKL. After 18 h, transfected cells were pretreated with or without ZVAD-FMK (5 µM) for 1 h before treatment with bortezomib (50 nM) for 24 h. After treatments, whole cell lysates were collected and then subjected to western blotting with indicated antibodies.

**
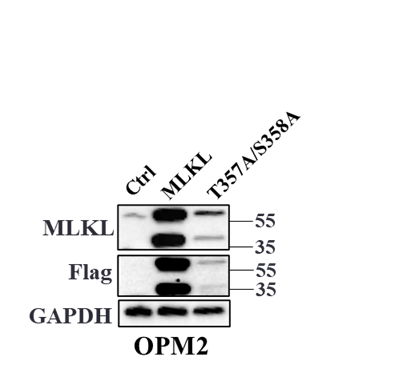
**

**Supplementary Figure 9.** RIPK3-dependent phosphorylation (Thr357/Ser358) is not required for MLKL cleavage in MM cells. OPM2 cells were transfected with 5 µg of vectors encoding C-terminal Flag-MLKLWT or C-terminal Flag-MLKLT357A/S358A. After 18 h, whole cell lysates were collected and then subjected to western blotting with antibodies against C-terminal of MLKL, Flag and GAPDH.


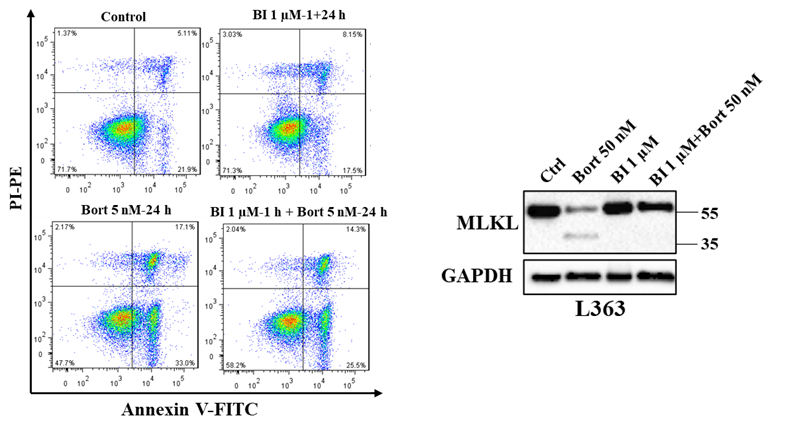


**
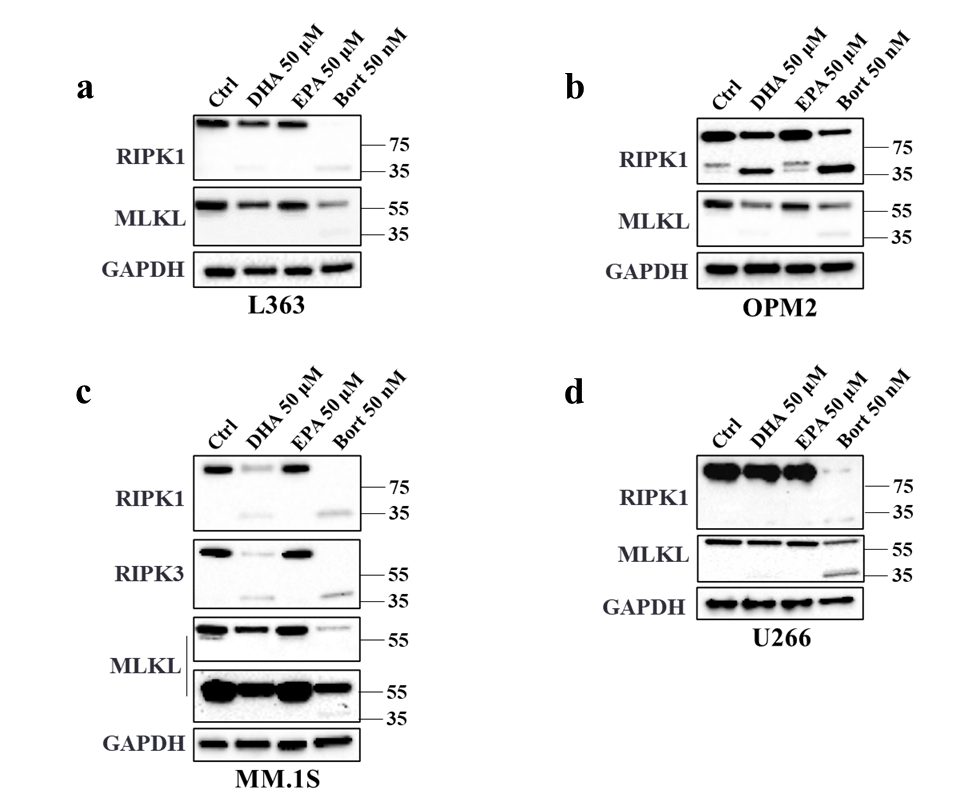
Supplementary Figure 10.** MLKL inhibitor BI-8925 inhibits bortezomib toxicity and bortezomib-induced MLKL cleavage in MM cells. L363 cells were pretreated with 1 µM of BI-8925 for 0 or 1 h and then incubated with bortezomib (5 nM) for 24 h. (a) Apoptotic cells were determined by Annexin V and PI staining. (b) After treatments, whole cell lysates were analyzed by western blotting with indicated antibodies.

**Supplementary Figure 11.** Cell death triggers induce RIPK1 cleavage in four MM cell lines. MM cells were treated with DHA/EPA (50 µM) or bortezomib (50 nM) for 24 h. After treatments, whole cell lysates were analyzed by western blotting with indicated antibodies.
